# Using Zebrafish to Study Multiciliated Cell Development and Disease States

**DOI:** 10.3390/cells13211749

**Published:** 2024-10-23

**Authors:** Thanh Khoa Nguyen, Sophia Baker, John-Michael Rodriguez, Liana Arceri, Rebecca A. Wingert

**Affiliations:** Department of Biological Sciences, University of Notre Dame, Notre Dame, IN 46556, USA; sbaker9@nd.edu (S.B.); jrodri28@alumni.nd.edu (J.-M.R.); lra63@cornell.edu (L.A.)

**Keywords:** ciliopathies, disease modeling, genetic interactions, multiciliated cell, kidney, pronephros, ependymal cell, nasal placode, zebrafish

## Abstract

Multiciliated cells (MCCs) serve many important functions, including fluid propulsion and chemo- and mechanosensing. Diseases ranging from rare conditions to the recent COVID-19 global health pandemic have been linked to MCC defects. In recent years, the zebrafish has emerged as a model to investigate the biology of MCCs. Here, we review the major events in MCC formation including centriole biogenesis and basal body docking. Then, we discuss studies on the role of MCCs in diseases of the brain, respiratory, kidney and reproductive systems, as well as recent findings about the link between MCCs and SARS-CoV-2. Next, we explore why the zebrafish is a useful model to study MCCs and provide a comprehensive overview of previous studies of genetic components essential for MCC development and motility across three major tissues in the zebrafish: the pronephros, brain ependymal cells and nasal placode. Taken together, here we provide a cohesive summary of MCC research using the zebrafish and its future potential for expanding our understanding of MCC-related disease states.

## 1. Introduction

Cilia are tubulin-based organelles that protrude from the cell and serve many important physical, chemical and physiological functions. Defects in cilia have been linked to many diseases that are well documented across the literature [1,2,3,4]. Historically, primary cilia have been widely studied as targets for many of those diseases. In recent years, however, there has been a surging interest in the field of studying MCCs and their relationship to ciliary-based conditions. MCC-related disorders are diverse, ranging from rare genetic conditions to chronic diseases that affect hundreds of millions of people around the world. Most recently, the rise of COVID-19, a global pandemic caused by the virus SARS-CoV-2, has continued to affect public health worldwide with its high transmission and mortality rate. Interestingly, recent reports have found a direct link between SARS-CoV-2 infection and defects in MCCs. Therefore, it is more critical than ever to implement simple and effective models to study MCCs. MCC studies have been performed with many tools, ranging from human cell lines to animal models [5]. However, despite many powerful advances in understanding MCC biology, there are many ongoing studies to address major open questions in the field: (1) What are the genetic mechanisms underlying MCC development? (2) What disease conditions are linked to MCC defects?

In this review, we explore recent reports that have advanced our understanding of these questions with a special focus on the use of the zebrafish model to study MCC development and disease. We discuss the processes behind MCC formation, including centriole amplification, basal body generation and docking, as well as the genetic factors involved in these events. Next, we provide an updated summary of many diseases across organs that have been linked to MCC defects, highlighting recent studies of the link between MCCs and SARS-CoV-2. We then explore the advantages of using zebrafish as a model organism for studying diseases, highlighting their effectiveness in modeling kidney diseases as a key example. Furthermore, we explore the abundance of MCCs in tissues in zebrafish, with a special focus on the pronephros, nasal placode and brain ependymal cells. Finally, we provide a comprehensive summary of the most current understanding of the genetic interactions that mitigate MCC ontogeny in the zebrafish.

## 2. General Structure and Formation of Cilia

A cilium is comprised of the basal body, the transition zone and the axoneme. The basal body is made up of centrioles, which act as a nucleating center to “grow” the cilium [6,7,8]. At the end of the basal body is the transition zone. This region is known to have about 100 proteins, which aid with the anchoring of the cilium. The transition zone also helps with ciliary-based signal transduction, which involves the trafficking of many molecules between the cilium and other cellular components [9]. Protruding from the cell is the axoneme, which exhibits a “9 + 0” arrangement, including nine peripheral microtubule doublets with similar length, which are arranged in a cylindrical fashion [10,11,12,13,14]. Motile cilia can exhibit this structure or can possess two inner microtubules inside the nine-microtubule ring, making up a “9 + 2” arrangement. The axoneme is produced and maintained through the process of intraflagellar transport (IFT), in which protein cargos are moved bidirectionally between the ciliary base and tip [15,16,17].

## 3. Centriole Formation and Biogenesis: The Basis of MCC Formation

**Figure 1:**
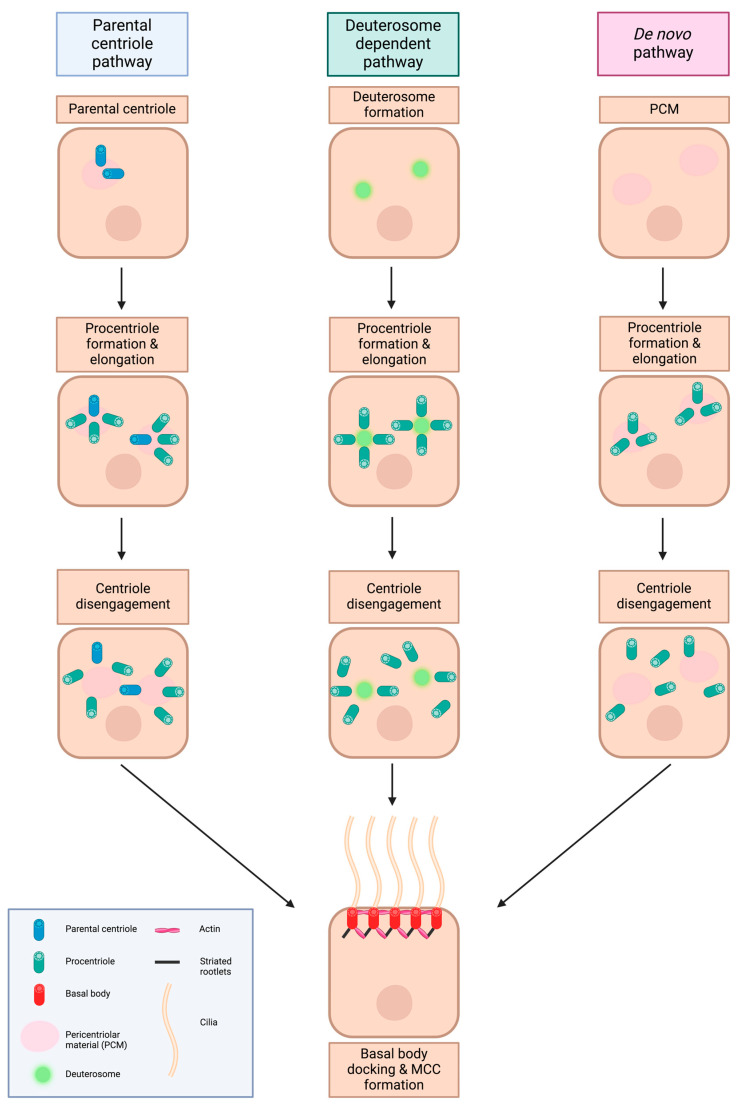
Different pathways of MCC formation. There are three known pathways of MCC formation: (1) Parental centriole pathway: the parent centriole serves as a template for two to eight centrioles arising from it. These centrioles will eventually mature, disengage and dock to the apical membrane to become basal bodies for MCC formation. (2) Deuterosome-dependent pathway: centrioles arise from the deuterosomes instead of parental centrioles. (3) *De novo* pathway: neither parental centriole nor deuterosomes are needed for centriole formation. The centriole is thought to arise and mature from the pericentriolar material cloud (PCM). Created with BioRender.com.

The process of centriole formation occurs in a precise sequence during the vertebrate cell cycle. Depending on whether cells are in the G1 or G2 state, they contain either two centrioles or two pairs of centrioles. New daughter cells inherit a pair of “disengaged” centrioles after mitosis and cilium formation begins in G0. However, if the cells continue to proliferate, they will undergo centriole duplication in the S phase, with daughter procentrioles “budding” from the parental centriole. The process of centriole elongation happens during the S and G2 phases. During the G2/M transition, more pericentriolar materials are accumulated, and two centrosomes become separated, forming mitotic spindles [18]. MCC progenitors include a centrosome with parental centrioles and a single non-motile primary cilium. When these progenitors proliferate, the cells ensure the presence of this centrosome-cilium combination by following the canonical centriole duplication pathway, forming new centrioles from pre-existing centrioles. After differentiation, MCCs become post-mitotic, and they produce another 30 to 500 centrioles. Eventually, these newly made centrioles will mature into basal bodies and serve as a template for ciliary outgrowth [19,20]. The process, termed centriole amplification, occurs in several ways (Figure 1). In one mechanism, termed the parental centriole or centriole-dependent pathway, the original parental centriole serves as template for about two to eight centrioles “sprouting” from the parental centriole. In the second mechanism, known as the deuterosome-dependent and the “acentriolar” pathway, deuterosomes are responsible for nucleating procentrioles. Deuterosomes are ring-shaped structures that can grow multiple centrioles from them [21]. Unlike the centriole-dependent pathway, this deuterosome-dependent pathway includes new centrioles that originate from deuterosomes. The centriole-dependent pathway is thought to produce 5% of the centriole population, while the deuterosome-dependent pathway is responsible for the other 95% [22,23,24,25,26,27]. In a third process of MCC generation, a *de novo* pathway, MCCs generate basal bodies without deuterosomes, as well as with a combined absence of both deuterosomes and parental centrioles. It was speculated that the pericentriolar material cloud could serve as a source of procentriole development and maturation [26,28].

## 4. Generation of Basal Body and Basal Body Docking

### 4.1. Centriole Assembly

Centrioles are important in proliferating cells and ciliogenesis. In proliferating cells, centrioles form the pericentriolar matrix, which becomes a centrosome. This pericentriolar matrix then nucleates and organizes the cytoplasmic microtubules. During G1 and G2, this centriole pair grows and organizes increased amounts of pericentriolar matrix, which helps recruit more microtubules when the cells enter mitosis. During ciliogenesis, the centrosome moves towards the cell surface, and the mother centriole of the centrosome is positioned in close proximity to the plasma membrane. The ciliary microtubules originate from the distal end of this centriole, thanks to the existence of the pinwheel-like distal appendage, a structure that helps anchor cilia to the plasma membrane [29]. In MCCs, this process is amplified by the recruitment of multiple simultaneously assembled centrioles [18]. After the generation of the centriole, basal bodies migrate to the MCC apical surface.

### 4.2. The Role of Actin Cytoskeleton in Basal Body Docking

Basal body docking is a complex process dependent on many genetic mechanisms. One of the major components in basal body docking to the apical membrane involves actin cytoskeleton development and genes associated with actin filament development. In early studies, basal body docking was dependent on actin filament assembly [30,31]. For example, cytochalasin D, an F-actin polymerization inhibitor, was studied using scanning electron microscopy (SEM) and transmission electron microscopy (TEM) to investigate its effect on basal body migration. It was found that basal body migration to the apical surface was inhibited, and that basal bodies anchored to the intracellular vacuoles instead [31,32]. Actin regulators, such as the phosphate loop ATPase Nubp1 or RhoA, also play an important role in this process [32,33]. Interestingly, planar cell polarity does not influence this anterior migration, but rather affects basal body orientation in the ependymal cells of the murine brain [34]. There are two distinct but interconnected pools of actin at the apical surface of MCCs: (1) a meshwork of actin cap at the apical membrane at the same plane as the basal bodies, creating a “net” in which the ciliary axoneme emerges from it and (2) a “subapical” pool of actin just below the cell surface [35].

Recently, it was reported that ELMO-DOCK1, a bipartite guanine nucleotide exchange factor complex for Rac1, and Ezrin, a membrane-cytoskeletal linker, play an important role in centriole/basal body docking. Zebrafish and *Xenopus* embryos deficient in Elmo and Dock1 were defective in basal body transport and docking. Additionally, ezrin morphants exhibited defective basal body docking and had a thinner apical actin network compared to controls. Furthermore, the ELMO-DOCK1 downstream effector Rac1 is known to be important for basal docking and spacing: rac1/rac1l double morphants exhibited increased basal bodies in cytoplasm and defective basal body transport [36].

Given the role of actin in trafficking basal bodies to the apical surface of ciliated cells, regulators of the actin network most likely also regulate basal body localization. However, this is not always the case. A previous study of the *cordon-bleu* (*Cobl*) gene found enriched expression in zebrafish tissues, in particular in the apical domain of ciliated cells near the apical actin cap. Morpholino models revealed an important role of *Cobl* in regulating apical F-actin, but reduction of *Cobl* expression did not disturb basal body localization [37].

### 4.3. The Role of Planar Cell Polarity (PCP) in Basal Body Docking

Another important component of basal body docking is the PCP pathway. The core PCP protein network includes the Cadherin EGF LAG seven-pass G-type receptor (Celsr1-3), Dishevelled (Dvl1-3), Frizzled (Fzd3, 6), Prickle (Pk1-2) and Van Gogh-like (Vangl1-2) [38]. Furthermore, after the polarity of PCP proteins, basal body docking is established and polarized immediately, and further refined after docking [39]. There are many important components that contribute to PCP signaling. For example, the PCP protein Vangl2 was detected at the base of the cilia [40]. The ciliary protein Inversin, with highly conserved ankyrin repeats and IQ domains, was found to interact with Akt at the basal body of primary cilia [41]. Additionally, the PCP protein Dishevelled (Dvl) plays an important role in basal body docking to the apical surface. Dvl localizes to the basal body, and Dvl morphants fail to dock the basal body properly. Furthermore, Dvl works with the PCP effector Inturned and the Rho GTPase for effective apical docking of the basal body [42]. In the MCCs of *Xenopus*, the PCP effectors Inturned or Fuzzy governed apical actin assembly and controlled orientation of cilia, thus playing a major role in the processs of basal body polarization [43]. Further, *Fltp*, a gene expressed in active PCP signaling within the MCCs of the lung and inner ear sensory hair cells, plays an important role in regulating basal body docking and positioning. *Fltp* KO mice exhibited basal body docking defects, and Fltp, the basal body positioning Dlg3 and PCP molecule Dishevelled 2 physically interacted surrounding the basal body region [44].

### 4.4. Other Genetic Components Important for Basal Body Docking

In addition to the actin cytoskeleton and PCP pathway, there are other genetic components important for basal body docking. For example, mutant cells without FoxJ1 failed to direct basal body positioning [45,46]. *Foxj1*-deficient mice also failed to dock basal bodies and lacked apical actin. In addition, FOXJ1 activates RhoA and RhoB and still exists when RhoA is inhibited, suggesting that FOXJ1 promotes RhoA in basal body docking and ciliogenesis [32]. Mice lacking *Foxj1* exhibited a decrease of calpastatin, an inhibitor of protease calpain and a decrease in the calpain substrate ezrin. Similar studies observed that *Foxj1*^−/−^ mice failed to have basal bodies anchor to the apical cytoskeleton [47].

Furthermore, in *Paramecium tetraurelia*, the lack of VFL3 and OFD1 causes deficient basal body docking, in which the lack of OFD1 results in defective assembly of the basal body distal part, while in VFL3-A deficient cells, the unanchored basal bodies had abnormal distribution of their associated rootlets [48]. Rootlets are cytoskeletal filaments located at the proximal end of a cilium that are thought to help orient basal body position and coordinate ciliary beating. Interestingly, defects in the basal body were also observed with *VFL3* in *Chlamydomonas* [49]. PtFOR20p, the *Paramecium* ortholog of FOR20, was found to be located at the basal bodies and required for making the transition zone, an important prerequisite for basal body maturation and docking. PtCen2p, an ortholog of Centrin 2, is important for recruitment of PtFOR20p at the basal body. PtCen3p, a Centrin 3 ortholog, is also important for basal body docking at the cell membrane [50]. Chibby (Cby), a 15-kD coiled coil protein conserved in all ciliated unikont animals, is important for ciliary vesicle formation and basal body docking to the apical cell membrane. Cby interacts with the distal appendage protein CEP164 when it is recruited to the distal centriole appendage. Cby forms a complex with CEP164 and Rabin8 to promote timely basal body docking to the apical membrane [51]. The tumor suppressor gene CYLD was also found to control apical docking of basal bodies in ciliated cells. Localization studies showed that CYLD is expressed at the centrosomes and basal bodies and forms a complex with centrosomal protein CAP350. Mouse *Cyld^Δ932^* mutants lack apical migration and docking of basal bodies. Furthermore, ciliogenesis was impaired by overexpression of CYLD in the cytosol or the presence of a catalytically inactive CYLD at the centrosome [52]. Further, C2 calcium-dependent domain containing 3 (C2cd3) was shown to be essential for ciliary vesicle docking in mammals, as it is required for recruitment of critical distal appendage proteins, and the absence of C2cd3 resulted in Ttbk2 not being recruited to the ciliary basal body, as well as failure to remove Cp110 from the ciliary basal body [53]. PtCen3p is a centrin isotype known to be participating in basal body positioning in *Paramecium* [54]. Assembly of the basal body appendage called the anterior left filament requires PtCen3p for efficient docking at the surface [55]. Lastly, the mother centriolar protein Cep164 is necessary for vesicular docking at the distal part of the mother centriole, interacting with the GEF Rabin8 and GTPase Rab8 in the process [56].

## 5. MCCs in Diseases: From Rare Conditions to Global Health Emergencies

**Figure 2:**
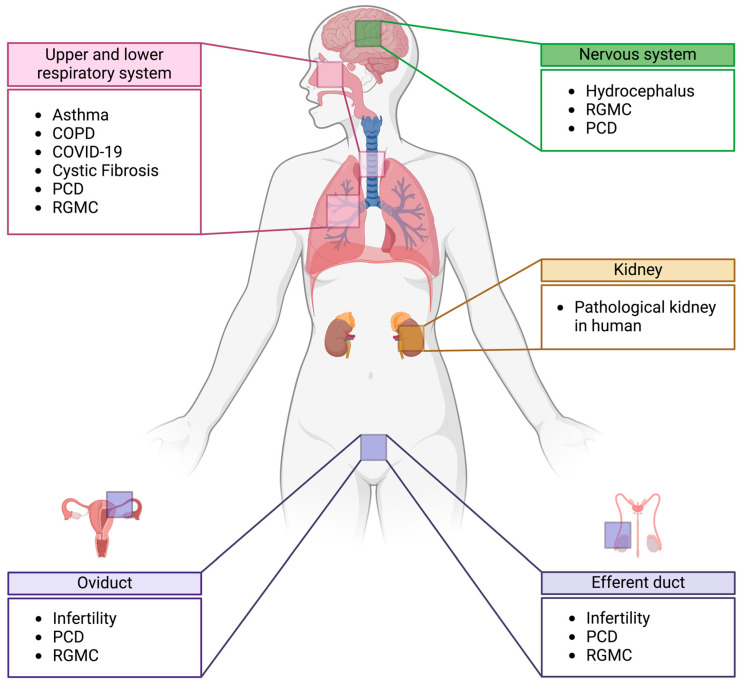
MCCs in human diseases. There are many pathological conditions across human tissues that are defective in MCC development and motility or have been linked to MCC-regulating genes. Pathological conditions involving MCCs were recorded in several tissues, such as the upper and lower respiratory systems, nervous system, kidney, oviduct and efferent duct. In the kidney, pathological conditions have been linked to ectopic development of MCCs. Created with BioRender.com.

The link between primary cilia and ciliopathies has been well documented [1,2,3,4]. Here, we focus on diseases that have been linked to defects in MCC development and motility, ranging from rare conditions to chronic diseases, as well as the recent global pandemic COVID-19 caused by the virus SARS-CoV-2 (Figure 2).

### 5.1. Primary Ciliary Dyskinesia (PCD) and Reduce Generation of Multiple Motile Cilia (RGMC)

PCD is a group of genetically heterogeneous disorders that are defined by dysfunctional beating of respiratory cilia, leading to deficient mucociliary clearance of the airways and chronic airway infections [57]. RGMC is one of the severe PCD phenotypes, involving similar pulmonary and fertility defects that are PCD-like. RGMC is a condition involving the reduction of MCCs across organs due to defects in ciliogenesis, leading to opportunistic infections [58,59]. Individuals with RGMC experience reduced mucociliary clearance along with upper and lower airway infections, postnatal respiratory distress syndrome, and hydrocephalus. However, no *situs* defects and nodal cilia defects have been observed in patients with RGMC, unlike individuals with PCD who have defects in both motile and nodal cilia [60]. Several studies found a link between RGMC and multiple genetic components that are crucial for MCC development. For example, *MCIDAS* is mutated in individuals with RGMC. Ultrastructural analysis using TEM revealed a reduction of cilia number and basal body misorientation. FOXJ1 and CCNO expression were abrogated in respiratory cells, suggesting that MCIDAS regulates both genes in the human respiratory tract MCCs [58]. Further, individuals with *CCNO* mutations exhibited a reduction in MCCs as well as defective centriole generation and basal body docking [59]. While *MCIDAS*, *CCNO* or *FOXJ1* deficiency affect MCC genesis, studies revealed some differences in the causes. Both *MCIDAS* and *CCNO* led to defects in centriole amplification in the deuterosome-dependent pathway, leading to a severely reduced number of basal bodies and thus a reduction in multiciliogenesis [61]. On the other hand, *FOXJ1* mutant individuals had a normal number of basal bodies per MCC, but many basal bodies were unable to dock to the apical membrane, leading to a reduction in ciliary number [62]. Additionally, NEK10, a protein kinase without defined function(s) in mammals, was shown to regulate MCCs in mucociliary clearance. *NEK10* deficiency led to reduced ciliary MCC formation and movement [63]. Researchers also determined that TP73, belonging to the TP53 family of transcription factors, is one of the contributors to MCC differentiation in RGMC. In *TP73* variants, it was observed that MCC numbers are reduced, together with decreased level of cell expression of FOXJ1 and RFX2, both of which are crucial multiciliogenesis transcription factors. Coupling with observed reduced functional ciliary airway clearance, these findings suggested that TP73 is a main player in RGMC [64].

### 5.2. COVID-19

For the past several years, COVID-19 has become one of the biggest public health concerns globally due to its high transmissibility and mortality. The symptoms of COVID-19 include difficult breathing, fever and dry cough, as well as headache, lethargy, difficult breathing, chest tightness, vomiting and diarrhea [65,66,67]. The SARS-CoV-2 virus is responsible for COVID-19 pathologies. ACE2, an angiotensin-converting enzyme, is a functional receptor for both SARS-CoV and SARS-CoV-2 [68,69,70]. In addition, TMPRSS2 and Furin are the proteases known to process the S-protein SARS-CoV to activate the virus and enable it to access the cytosol [71,72]. Recent evidence showed higher susceptibility of SARS-CoV-2 infection with the TMPRSS2-expressing cell line, suggesting a crucial role of TMPRSS2 in SARS-CoV-2 infection [73]. Interestingly, both ACE2 and TMPRSS2 were found to be expressed in MCCs of the nasal epithelium. ACE2 expression increases with age in MCCs [74,75]. Indeed, it was later revealed that in the course of infection, SARS-CoV-2 protein level is high and localized specifically to the epithelial MCCs but not the goblet cells of the nasal respiratory epithelium. Additionally, the primary target of SARS-CoV-2 replication is at MCCs but not secretory or basal cells [72,76,77,78]. Consequently, SARS-CoV-2 infection led to a rapid decrease in MCCs, and infected MCCs also suffered structural defects such as axoneme loss and basal body misorientation, and ciliary function in mucociliary clearance was severely impaired. Furthermore, SARS-CoV-2 also led to an inhibition of the MCC regulator Foxj1 [78]. Taken together, these findings highlight the importance of further studies as to how SARS-CoV-2 infection impacts MCCs.

### 5.3. Chronic Obstructive Pulmonary Disease (COPD)

Mucociliary clearance is an important process to move fluid, pathogens and particles that enter the airways. This process is driven by the proper beating of MCCs [79]. COPD is one of the deadliest chronic diseases of the respiratory system involving the failure of robust mucociliary clearance. The disease is characterized by progressive airflow limitation, combined with increased inflammatory responses in the lung as a reaction to toxic gases or harmful particles [80]. The incidence rate of COPD is higher in males and is highly associated with smoking history [81,82]. Indeed, cigarette smoking is one of the major risk factors of COPD, as smokers are exposed to a combination of reactive oxidants, heavy metals and human carcinogens in cigarette smoke [83]. Indeed, heavy smoking impairs mucociliary clearance [84]. Intriguingly, studies found a link between COPD and MCC development and motility. In earlier studies, cilia were shorter in the airway epithelium of healthy smokers compared to non-smokers and even shorter in COPD smokers compared to both healthy smokers and nonsmokers [85,86]. Through more recent investigation, MCC number was found to be decreased in fully differentiated COPD epithelia compared to non-COPD respiratory epithelia in air-liquid interface cultures. MCC regulators such as GMNC, MCIDAS, FOXJ1 and RFX2/3 were also downregulated in COPD vs non-COPD [87]. A decrease in FOXJ1 and miR-449a, an essential miRNA of MCC development [88,89], has also been observed with cigarette smoke exposure [83]. In the airway epithelium, the number of MCCs decreased, while the number of primary ciliated cells increased [90]. In addition, reports showed that COPD impairs MCC motility. Cilia beating frequency (CBF) decreased in subjects with moderate to severe COPD [91], which was found to include the bronchial regions [92]. More specifically, CBF in MCCs decreased in COPD patients compared to non-COPD patients [90].

### 5.4. Cystic Fibrosis (CF)

CF is a disease caused by the mutation in the cystic fibrosis transmembrane conductance regulator (CFTR) ion channel [93]. This channel helps control chloride secretion and ultimately influences the function of other membrane proteins such as the epithelial sodium channel [93,94]. CFTR defects lead to an increase in fluid absorption at the epithelial surface, which results in a build-up of thick mucus in a variety of organs, such as the lung, pancreas and reproductive organs. In the respiratory tract, this thickening layer of mucus compresses the periciliary layer, thus slowing down and paralyzing cilia function in mucus clearance, creating opportunities for repeated infections [95]. Interestingly, TEM studies have correlated abnormal ultrastructural changes in respiratory MCCs of CF patients with advanced clinical inflammation [96,97]. Specifically, patient samples displayed abnormal microtubule doublets as well as single peripheral microtubules replacing normal doublets. Further, ciliated cells exhibited a dearth of dynein arms, suggesting MCC motility would be compromised, and only a focal presence of ciliated epithelium was found, consistent with MCC attrition [96]. Expression of *CFTR* is low in MCCs compared to its expression in ionocytes in the proximal airway, which suggests a more central role of ionocytes in CFTR activity in the airway epithelium [98,99]. However, the association of structural changes in the airway epithelium suggests that a component of the pathophysiological progression of disease impacts MCC form, and thereby their functionality.

### 5.5. Asthma

Asthma is a serious respiratory disease affecting hundreds of millions of people in the world. It is characterized as an inflammatory disease that leads to obstruction, overproduction of mucus, airway responsiveness and airway wall remodeling [100]. Common symptoms include shortness of breath, coughing, wheezing and chest tightness [101]. Interestingly, a study found dysfunctional cilia activity in individuals with severe asthma characterized by decreased CBF, higher dyskinesia and motility indices. A reduction in ciliated cell number and ciliary disorientation was also observed [102]. It was reported that the T helper 2 cytokine, IL-13, experienced an elevated level in individuals with asthma [103,104]. Interestingly, IL-13 was found to repress MCIDAS and FOXJ1 expression independent of Notch signaling but dependent on the activation of the JAK/STAT signaling instead [105]. These findings suggest a promising connection to explore between asthma and MCCs and MCC-regulating genes in future studies.

### 5.6. Hydrocephalus

Hydrocephalus is defined by the enlargement of the cerebrospinal fluid (CSF)-filled brain ventricles. This enlargement of brain ventricles is due to several reasons, such as reduction in fluid reabsorption, anatomical obstruction or increased CSF production [106]. Hydrocephalus compresses the brain, leading to increased intracranial pressure, eventually causing neurologic decline and death in extreme cases [107]. Cilia defects have long been cited as an essential player in hydrocephalus, given their importance in fluid propulsion [108,109,110]. In particular, several studies found MCCs and MCC-regulating genes to play a major role in hydrocephalus formation [111]. For example, there was a 10% increase in hydrocephalus in humans with a *CCNO* mutation [57]. Mice deficient in *Ccno* also display hydrocephalus [112]. Hydrocephalus was also detected in individuals with biallelic *MCIDAS* mutations [58,113]. In addition, heterozygous *de novo* mutation in *FOXJ1* resulted in hydrocephalus in humans [62]. While hydrocephalus was detected in both PCD [114] and RGMC in humans [57,58,59], hydrocephalus is more frequent in individuals with RGMC. These findings suggest that multiciliogenesis defects play an important role in hydrocephalus development.

### 5.7. Pathological Kidney States

Mammalian kidney epithelial cells possess a single non-motile primary cilium with the 9 + 0 pattern [115,116]. However, MCCs exist in the human fetal kidney and patients with different renal disease states [116,117,118,119]. Recently, MCCs in human kidney pathologies have been more thoroughly studied, where MCCs were found to localize to the proximal tubule segment and to express FOXJ1 and RFX3, transcription factors of motile cilia. These findings suggest that MCCs emerge in multiple kidney pathologies [120].

### 5.8. Infertility

Infertility is one of the major common ciliopathies in the reproductive tracts of both males and females that has been linked to MCC dysfunction. In the male reproductive tract, the efferent ducts (EDs) contain MCCs in the epithelium and facilitate sperm movement from the testis to the epididymis [121]. There, MCCs serve an important rotational motion to stir luminal content to provide consistent turbulence, thus preventing sperm aggregation [121,122]. Without MCCs, sperm aggregation can cause ED obstruction, which can lead to male infertility [123]. In the female reproductive tracts, MCCs are found in the oviduct, especially at the infundibulum—the portion within closest distance to the ovary, where ovulation transpires [124,125]. The transport of oocytes is essential for fertility and believed to be achieved through a combination of smooth muscle contraction, motile cilia beating and flow of tubal fluid [126,127,128].

Defects in MCC-regulating genes have been shown to cause infertility. For example, in males, *e2f5* zebrafish mutants failed to form MCCs in the nasal placode and pronephric ducts, and spermatogenesis was arrested [129]. Similarly, mutant mice deficient in *E2f4* and *E2f5* were sterile due to complete loss of MCCs in the EDs [130]. *Gemc1* mutant male and female mice were infertile, and the oviduct of female *Gemc1* mutants lacked MCCs, as well as the expression of FOXJ1 and RFX3 in the oviduct compared to WTs [131]. Female *Mcidas* mice mutants lacked oviduct MCCs and were infertile [132]. Furthermore, male mice without *Gemc1*, *Mcidas* and *Ccno* experienced severe reduction in MCCs in the ED, and consequently spermatozoa agglutination and infertility [133]. Both male and female mice deficient in *Ccno* were infertile, and fewer cilia were present in the oviducts [134]. Human MCCs were also investigated in both sexes in cases of PCD and infertility: a male patient with an *MCIDAS* mutation and a female patient with *CCNO* mutation. Both patients displayed abnormal MCCs in their EDs/oviducts and reduced numbers of basal bodies [135]. Further, genetic ablation of *miR-34b/c* and *miR-449a/b/c* caused MCC defects in the EDs and infertility [121]. Mice deficient in *DNAH5* (*Mdnah5*^mut/mut^) have defective ED cilia motility, and humans with *DNAH5* defects display reduced sperm numbers [136]. The distal appendage protein CEP164 also plays a role in ED multiciliogenesis, in which mice lacking CEP164 displayed absence of MCCs in the EDs, leading to sperm agglutination and aggregation and ED obstruction, leading to infertility [137].

## 6. Zebrafish as a Model for MCC Development and Disease

### 6.1. Why Is Zebrafish a Good Model to Study MCC Biology?

**Figure 3:**
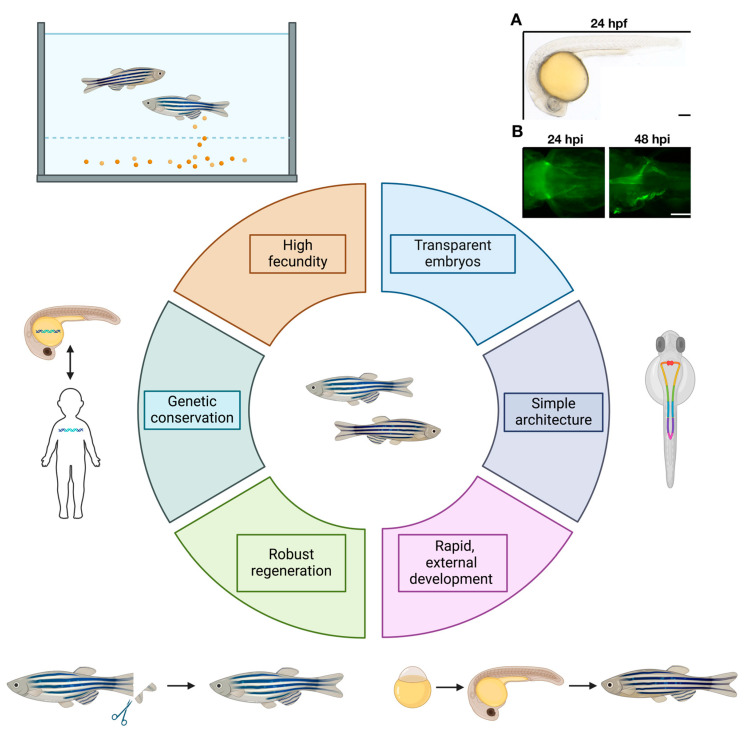
Zebrafish as a model to study development and disease. Zebrafish host many important traits, allowing them to be a great biomedical model to study embryogenesis and model disease states. (1) Transparent embryos: live zebrafish embryos are optically transparent and can be maneuvered to maintain transparency, allowing for development studies. (**A**) The zebrafish embryo at 24 hpf with almost no pigment across the body. Scale bar = 100 μm. (**B**) Graph depicts the migration of fluorescent 40 kDa Dextran conjugate in the proximal tubule of live embryo from 24 h post-injection (hpi) to 48 hpi. Scale bar = 100 μm. (2) Simple architecture: zebrafish have simpler organization than other animal models, while still maintaining complexity. For example, the zebrafish pronephros contains only two nephrons while being fully segmented, allowing for effective renal studies. (3) Rapid, external development: zebrafish development and organogenesis happen rapidly, allowing for organogenesis studies. (4) Robust regeneration: zebrafish can regenerate many tissues, allowing for recovery and regeneration studies. (5) Genetic conservation: zebrafish share about 70% of their genes with humans, allowing for studies of genetic diseases. (6) High fecundity: zebrafish are highly productive and can breed year-round in good conditions. Created with BioRender.com.

Multiple systems have been used to study MCCs, including *ex vivo* culture from human samples to animal models such as mouse, *Xenopus* or zebrafish [5]. Zebrafish offers several important benefits that complement the use of other models. For example, *ex vivo* culture from human samples can generate brain and choroid plexus (ChP) organoids, but so far, only primary cilia have been described [138,139]. Meanwhile, MCCs have been detected in several locations, including the ChP in the zebrafish brain [140]. Overall, zebrafish have several benefits for MCC development and disease studies (Figure 3). First, zebrafish embryos develop externally and are transparent. These traits allow researchers to easily observe the physiological mechanisms of development in real time without intensive manipulation [141,142,143,144]. For example, dextran-FITC clearance can be used to assess MCC motility in driving renal clearance in real time: it utilizes a fluorescent sugared molecule to track its migration across time points. Furthermore, zebrafish undergo rapid organogenesis, as illustrated by the fact that within 24 h post fertilization (hpf), the heart is functional, and a primitive circulatory system has formed along with the embryonic kidney [145,146].

Beyond gross physiological observations of development and disease in zebrafish, this model has been utilized as a relevant genetic model for better understanding vertebrate gene function [147,148]. The zebrafish genome is approximately 70% conserved with the human genome. Interestingly, about 82% of disease-related genes in humans are conserved in the zebrafish as well [144,148]. The ability to trace human genetic functions and disease in zebrafish, in conjunction with the aforementioned physiological aspects, makes zebrafish a relevant model for understanding human genetic diseases [149,150]. Interestingly, many zebrafish tissues, such as the kidney, have a much simpler architecture yet still maintain a high level of similarity in terms of tissue specification and genetics to mammalian counterparts [150,151].

Kidney research is one example where the zebrafish are useful as a model organism because they have a simple but conserved architecture of nephron functional units. Human kidneys contain approximately 900,000 to 1 million nephrons [146,152]. The zebrafish embryonic kidney contains two nephrons that form within 24 hpf [150,153,154,155]. The segmentation of these nephrons into proximal and distal regions largely recapitulates the organization of the human nephron [156,157]. There are differences, such as zebrafish nephrons lacking a loop of Henle, which is used for water conservation in other species [158]. The presence of renal MCCs in the embryonic kidney provides an avenue to study the genetic pathways that pattern this lineage.

### 6.2. Multiciliated Tissues in Zebrafish

Many model systems have been used to study MCCs, including *ex vivo* cultures from human samples to animal models such as mouse, *Xenopus* or zebrafish [5]. Zebrafish offer several important benefits that complement the use of other models. For example, *ex vivo* culture from human samples can generate brain and choroid plexus (ChP) organoids, but so far only primary cilia have been described [138,139]. Meanwhile, MCCs have been detected in several locations, including the ChP in the one-month-old zebrafish brain [140]. Zebrafish have several benefits over rodents for disease modeling, such as smaller size, higher number of offspring, rapid and external development, longer lifespan and most notably easier genetic manipulation and drug administration, allowing for the potential to create experimental models to test personalized medical therapies [141].

#### 6.2.1. Pronephros

**Figure 4:**
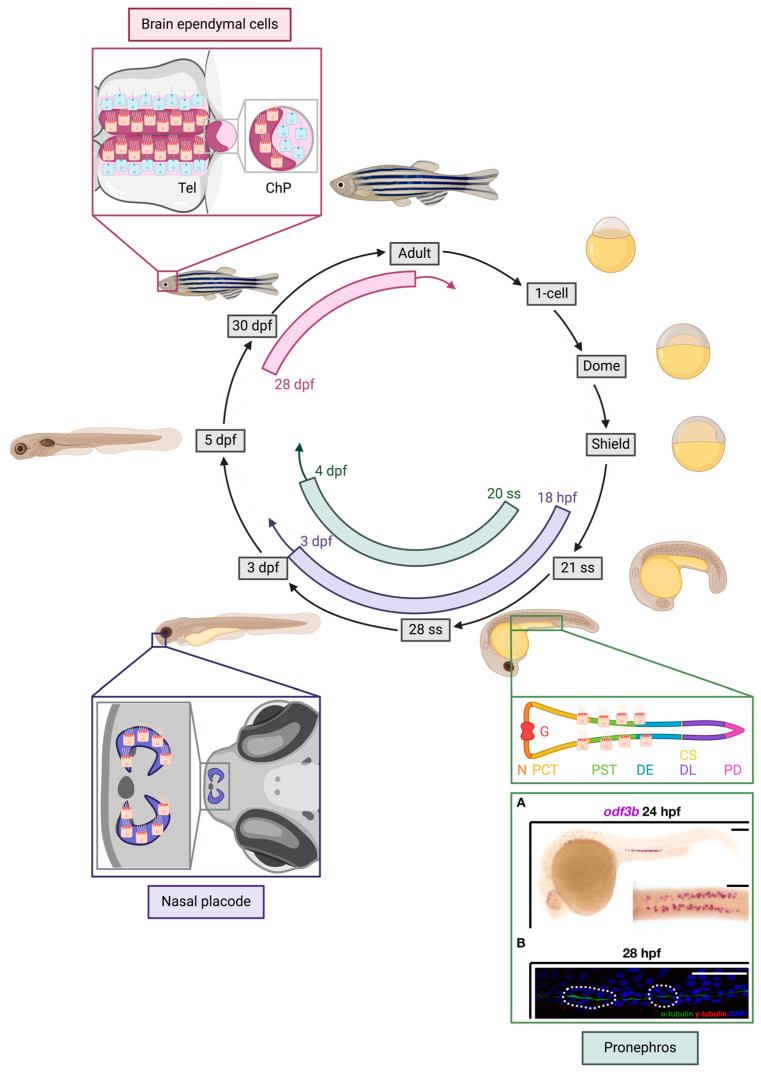
MCC tissues in the zebrafish. MCCs appear in many tissues in the zebrafish, such as the brain ependymal cells, nasal placode and pronephros. (1) Pronephros: the zebrafish pronephros is segmented into the glomerulus (G), neck (N), proximal convoluted tubule (PCT), proximal straight tubule (PST), distal early (DE), corpuscles of Stannius (CS), distal late (DL) and pronephric duct (PD). At 24 hpf or 28 ss, MCCs are distributed in a salt-and-pepper manner from the caudal end of the PCT to the rostral region of the DE. MCCs were detected as early as 20 ss. (**A**) 24 hpf WISH with MCC marker *odf3b* to demonstrate MCCs. Scale bar = 100 μm, inset = 50 μm (**B**) 28 hpf whole-mount immunofluorescence for acetylated α-tubulin (cilia, green), γ-tubulin (basal bodies, red) and DAPI (nucleus, blue) in the proximal pronephros of WT embryos. The white dash depicts MCC bundles. Scale bar = 50 μm. (2) Nasal placode: MCCs are located in the lateral rim of the nasal placode. The signal of Gmnc, the master regulator of MCC formation, was detected in the nasal placode as early as 18 hpf. (3) Brain ependymal cells: MCCs are detected around 28 hpf and highly enriched near the midline of the tela choroida, the epithelial layer above the dorsal telencephalon (Tel) and forebrain choroid plexus (ChP). Colored bands depict the time range in which MCCs were reported in each tissue in literature. Created with BioRender.com.

Since the embryonic zebrafish nephrons contain MCCs, this provides an opportunity to study MCC development. The MCCs are interspersed in a peppered fashion with monociliated epithelial cells in the PCT, PST and DE segments (Figure 4). Maturing MCCs are found within these segments as early as the 24 hpf stage. The appearance of MCCs progenitors can be detected as early as the 20 somite stage (ss), and by the time embryos reach 96 hpf, more than 60 MCCs can be detected [8,159,160]. Within these segments, MCCs are required for proper fluid propulsion during the production of urine [161].

#### 6.2.2. Nasal Placode

Besides pronephros, MCCs are highly present in the nasal placode, also known as the olfactory placode. For example, *rfx2* is a known marker to label MCCs in the pronephros [162,163]. Interestingly, *rfx2* is also highly expressed in the nasal placode, together with other factors highly expressed in MCCs such as miR-34b and *cmyb* [164]. Gmnc, the master regulator of MCC formation, was also detected in the zebrafish nasal placode as early as 18 hpf, and *gmnc* mutants exhibit a loss of the prominent MCCs in this region [165]. Various studies detected MCCs at the lateral rim of nasal placode at various time points between 48 and 72 hpf [164,166,167,168] (Figure 4). The MCCs of the zebrafish nasal placode are likely ciliated olfactory sensory neurons, which play a significant role in detecting odors for behavioral choices such as escaping from predators or finding food. They express olfactory receptors that help differ between specific odor environments [169]. Interestingly, several respiratory MCC-related conditions, like asthma, COPD and COVID-19 have been associated with a loss of scent in patients [170,171,172,173,174]. Therefore, understanding zebrafish nasal placode MCC development may also open a promising path to model the sensory aspects of human respiratory diseases.

#### 6.2.3. Brain Ependymal Cells

Cerebrospinal fluid (CSF) is the fluid produced by ependymal cells of the choroid plexus (ChP). CSF exists inside the brain and spinal cord and plays an essential role in the homeostasis of the central nervous system [175]. Motile cilia, including motile MCCs, are crucial for CSF fluid flow, and defects in MCCs have been linked to buildup of fluid in the brain, causing hydrocephalus in zebrafish [176]. The flow of CSF is dependent on the ependymal cilia in the nervous system. Previous studies classified ependymal cells into three groups, based on the number of cilia on their surface. The majority of ependymal cells are motile MCCs with about 20–100 cilia per cell, called E1 cells. A second group of ependymal cells are motile and bi-ciliated, called E2 cells, composing only 5% of the ventricular surface and more commonly found in the third ventricle, cerebral aqueduct and fourth ventricle [109,177,178]. A uniciliated population termed E3 cells is also located in the third ventricle. E3 cells possess primary cilia with a 9 + 0 microtubule structure [177]. In the zebrafish brain, MCCs emerge around 28–32 days post-fertilization (dpf) at the location of the tela choroida, the epithelial layer above the dorsal telencephalon (Tel) and the forebrain ChP, especially in the midline of dorsal telencephalon and anterior forebrain ChP (Figure 4). By adulthood, MCCs cover a large portion of the dorsal telencephalon [140].

### 6.3. Disease Modeling in Zebrafish: A Future Horizon for Studying MCC Related Conditions

Zebrafish have proven to be a powerful model for many human diseases. For example, zebrafish have been used as amenable cancer models thanks to the development of mutations via transgenic lines or chemical carcinogens [179]. Some of the cancers that have been studied in zebrafish include melanoma, pancreatic adenocarcinoma and leukemia [180,181,182]. The high degree of neurological and behavioral similarity between zebrafish and humans also makes them excellent candidates for studying brain disorders such as Alzheimer’s disease or spinocerebellar ataxia [183,184]. Additionally, zebrafish have been used to study bone diseases due to beneficial aspects such as the ability to do real-time live imaging of skeletal development and repair. Some of the bone diseases modelled by zebrafish include arthritis, osteogenesis imperfecta and osteoporosis [185,186,187]. In cardiovascular diseases, zebrafish have been well utilized, notably with the use of the *silent heart* (*sih*) model to study molecular pathways involved in cardiac disorders or studies of the *kcip1* gene, which is strongly associated with atrial fibrillation [141]. In addition, zebrafish have been used as a model to study several kidney diseases, such as diabetic neuropathy using the elmo1 mutant model or polycystic kidney disorders [188,189].

Interestingly, given the widespread use of zebrafish to model diseases, progress in modeling MCC diseases using the zebrafish model has been nascent. Among ciliopathies, zebrafish have been increasingly utilized to study several known ciliopathies. For example, the zebrafish model of scoliosis has been long studied, and recent work revealed a promising relationship between cilia defects and onset of scoliosis [176,190,191]. In addition, several other ciliopathies, such as left-right organ patterning, retinal diseases, polycystic kidney, and reproductive diseases have been modeled using the zebrafish [192]. In the kidney, an ongoing stream of research has identified important genes in MCC development using the zebrafish kidney [165,193,194,195,196].

## 7. The Genetics of MCCs: Genetics Mechanisms Governing MCC Development and Motility Across Zebrafish Tissues

**Figure 5:**
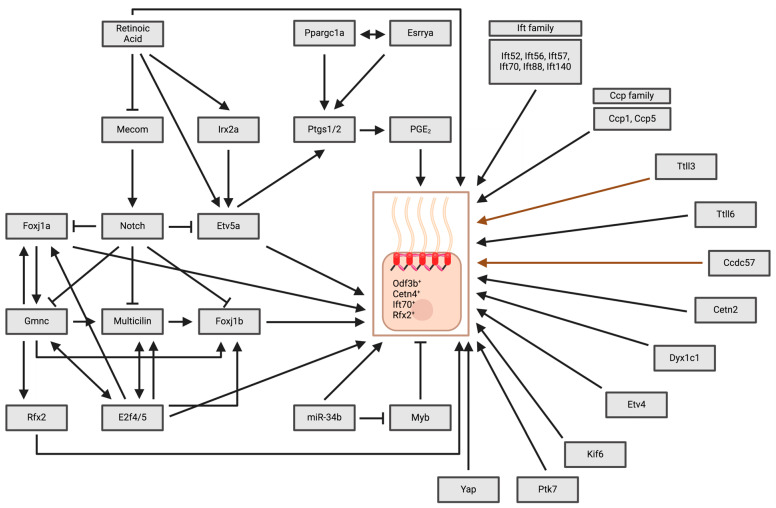
Genetic map of MCC development across zebrafish tissues. Map showing genetic interaction across different genetic factors in MCC development that were reported in the zebrafish. Interactions include activation (black arrows), inhibition (black inhibition arrow) and cooperation between factors (black double-headed arrows). Brown arrows denote factors that have been shown to influence MCC organization. Created with BioRender.com.

**Figure 6:**
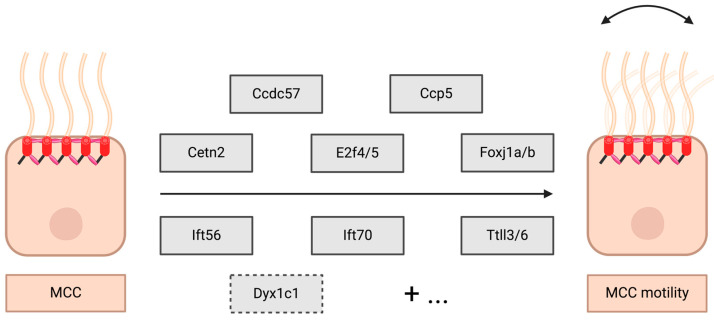
Essential genetic factors for MCC motility in zebrafish. Map showing genetic factors that were reported in literature to help regulate aspects of MCC motility, such as beating pattern, amplitude or frequency in zebrafish. Boxes denote factors that were shown to be essential in MCC motility. Dashed box denotes a factor that was found to be important for dynein arm development and potentially important for MCC motility. Created with BioRender.com.

A growing body of research has built our understanding of the genetic interactions that regulate MCC ontogeny in the zebrafish. In this section, we will provide a comprehensive summary of the genetic mechanisms that govern MCC development across three major MCC tissues in zebrafish: pronephros, brain ependymal cells and nasal placode (Figure 5). We will also provide details on genetic factors that were reported to contribute to MCC motility in zebrafish (Figure 6). We begin with Notch signaling, which is held to mark the beginning of MCC specification, followed by discussion of key Notch targets that have been found to date. We then discuss several other genetic pathways that have been shown to dictate MCC patterning and differentiation in various tissues.

### 7.1. Notch Signaling and Its Downstream Genetic Targets

#### 7.1.1. Notch Signaling Lateral Inhibition

Notch signaling directs MCC development within zebrafish tissues via the lateral inhibition mechanism, a process that has been implicated in controlling cell differentiation in other cell populations as well [197,198,199,200]. Notch lateral inhibition has been known to cause cells to enter a regulatory loop that causes neighboring cells to differentiate into different fates [201]. Activation of Notch helps silence the ciliation process in cells [202]. Notch inhibition leads to an increase in MCCs at the expense of other cell types in zebrafish—a phenomenon seen in various models, including human airway epithelium and *Xenopus* development [88,203]. In the zebrafish, Notch signaling controls left-right asymmetry and cilia length in Kupffer’s vesicle (KV) [204]. Regarding MCCs, a study found the Notch ligand Jagged2 to be expressed in MCCs. Additionally, embryos deficient in *jagged2* or the Notch receptor *notch3*, as well as the *mind bomb* mutants, which lack the E3 ubiquitin ligase important for Notch signaling [205,206], experienced an expansion of MCC-positive markers such as *shippo1/odf3b* and *rfx2* at the expense of the reduction in the expression of ion transporters such as *trpm7* and *slc13a1*. Similarly, inhibition of gamma-secretase by DAPT to block Notch cleavage [207] also caused expansion of MCC numbers and a reduction in ion transporter populations. Conversely, heat-shock induction of Notch1a ICD caused reduction in MCCs. Further, this study showed that Jagged 3 and Notch signaling is important for MCC development and the switch between MCC/transporter cells in the pronephros [162]. The Jagged2a-Notch1a/Notch3-Her9 system was also found to be important to facilitate the differentiation between MCCs and primary ciliated cells in the pronephros [163]. Mirroring these findings, blocking Notch signaling causes an increase in nasal MCCs and was associated with an expansion of *foxj1a* and *foxj1b* throughout the nasal epithelium [208].

#### 7.1.2. Geminin Coiled-Coil Domain-Containing Protein 1 (Gmnc)

Gmnc and Mcidas are factors downstream of Notch signaling that play an important role in MCC differentiation across species in zebrafish along with *Xenopus* and mice [131,165,209,210]. Gmnc was observed in several MCC tissues, and *gmnc* morphants and CRISPR-generated mutants formed kidney cysts and lacked MCCs in the kidney tubules. Researchers found that Gmnc is downstream of Notch but upstream of Mcidas in MCC specification, as *mib* embryos injected with *gmnc* MO did not have *mcidas* expression. Interestingly, *mib* mutants with *gmnc* MO also showed abrogated expression of *foxj1b* and *rfx2*, while expression of *foxj1a* remained. *gmnc* was identified as a target of FoxJ1a, and overexpression of *foxj1a* led to an increased level of *gmnc* [165]. Gmnc expression was also highly expressed in one-month old zebrafish at locations with high MCC population in the brain, such as the anterior forebrain ChP and TC. Intriguingly, *gmnc* mutants at the adult stage displayed an absence of MCCs compared to WT [140].

#### 7.1.3. Multicilin (Mcidas)

Multicilin, encoded by the *mcidas/Mcidas/MCIDAS* gene, is a small coiled-coil protein that has been known to be essential for MCC differentiation in various models, such as *Xenopus* and the human airway [209,211]. The protein is located in a conserved area in the chromosome essential for MCC differentiation. On one side, *mcidas* is flanked by *ccno*, a critical gene for RGMC, and on the other side by *cdc20B*, a gene encoding for miR-449a/b/c, which has also been known to be important for MCC differentiation [59,88,211]. *mcidas/Mcidas/MCIDAS* lacks a DNA-binding domain; therefore, its mechanism is unknown. Multicilin has a motif of 45 amino acids called the TIRT domain, which plays an essential role in MCC specification. Multicilin plays an essential role in the process of centriole biogenesis, which is essential for the massive centriole assembly required for differentiating MCCs. Multicilin forms a tertiary complex with E2f4/5 and Dp1, activating genes required for centriole biogenesis [211]. Multicilin has been found to regulate MCCs in human and *Xenopus* skin as well as mouse tracheal epithelial tissues [58,209].

In zebrafish, Multicilin has also been established to be an essential regulator of MCC differentiation. Multicilin is expressed in the developing MCCs of the pronephros. Multicilin loss of function did not show a major effect on MCC formation in zebrafish kidney tubules. In *mib* mutant embryos deficient of Notch signaling, *mcidas* expression increased, suggesting that Notch inhibits *mcidas* activity [165]. *mcidas* morphants exhibited less *foxj1b^+^* cells in the pronephros, suggesting *foxj1b* functions downstream of *mcidas.* Additionally, Multicilin interacts with E2f4 and E2f5 and has a synergetic effect with E2f5 in inducing ectopic MCCs. The study concluded that Multicilin activity is not obligatory for MCC specification in zebrafish [161]. Interestingly, studies of *Xenopus* and humans demonstrated a more important role in MCC formation, suggesting that there might be differences in the essentiality of Multicilin in MCC formation across species [58,209].

#### 7.1.4. The E2f Transcription Factor Family: E2f4 and E2f5

The E2f transcription factor family is essential for MCC development. The E2f transcription factor family has been known to be important for regulating cell cycle, apoptosis and transcription [212]. They are divided into different categories, including activators, repressors and inhibitors [129,213]. The MCC activators Gmnc and Mcidas form a complex with E2f4/5 and Dp1 to control the transcriptional program of MCCs [131,168,210,211]. In the zebrafish, researchers found that E2f5 played an important role in spermatogenesis and MCC differentiation. *e2f5* zebrafish mutants not only developed gametogenesis defects but were also deficient in MCCs in the nasal placode and pronephric ducts. Additionally, E2f5 activates *jag2b* to inhibit MCC fate [129]. Furthermore, E2f5 deficiency is associated with a lack of expression of *mcidas* and *foxj1b* in the pronephros and absence of *foxj1a* expression in the nasal placode of *e2f4*^+/−^; *e2f5*^−/−^ embryos [168]. In addition, E2f5 has a more important role in MCC development than E2f4, in which *e2f5* mutants experienced abrogation of MCCs in the pronephros and a reduction in MCCs in the nasal placode, while MCCs were unaffected in *e2f4* mutants. Interestingly, *e2f4*; *e2f5* double mutants showed a drastic reduction in both MCCs in the pronephros and nasal placode, and only single cilia were present in pronephros. Furthermore, in *e2f4*; *e2f5* double mutants, cilia movement was uncoordinated in the pronephros. These findings suggest that E2f4 and E2f5 have overlapping functional roles in MCC development in the nasal placode as well as the pronephros, with E2f5 playing a more substantial role [129,168].

#### 7.1.5. The Rfx Transcription Factor Family

The Rfx family of transcription factors is known to be essential for regulating ciliogenesis across animal models [162,163,214,215,216]. Among the Rfx transcription factors, Rfx2 and Rfx3 are essential for MCC development. RFX3 was found to co-activate FOXJ1 in human airway epithelial MCCs [217]. In the zebrafish, *rfx2* and *shippo1* have been found to colocalize with MCCs in the pronephros, and they are highly expressed in the nasal placode as well [162,164]. Additionally, Notch signaling has been shown to play an important role in MCC development by regulating *rfx2* [162,163]. The zebrafish *rfx2* gene is homologous to the *daf-19* gene, an important gene that controls cilia formation in the nematode *C. elegans* [214]. Much remains to be known about the role of other Rfx transcription factors in MCC development in zebrafish tissues.

#### 7.1.6. Foxj1

Foxj1 is a winged helix transcription factor important for cilia formation in the nasal respiratory epithelium in mice [218] and for differentiating progenitor cells into respiratory MCCs in human and *Xenopus* [217,219]. Foxj1 was also found to be essential for MCC development in *Xenopus* larval skin [220]. Zebrafish express two *foxj1* genes, *foxj1a* and *foxj1b* [221]. In the zebrafish, Foxj1 is expressed in various tissues, such as the floor plate, forerunner cells, KV, pronephric duct and the developing kidney [222]. Both genes are strongly expressed in multiple zebrafish ciliated tissues, where Wnt/β-catenin was found to regulate Foxj1 expression and cilia formation and motility in the zebrafish KV [223]. For example, *foxj1a* is highly expressed in the zebrafish nasal placode, and both *foxj1a/b* are expressed in MCCs in the pronephros. Interestingly, *foxj1a* knockdown resulted in paralysis of nasal placode motile cilia. *foxj1a* and *foxj1a/foxj1b* double knockdown impaired MCC motility in the nasal placode, and *foxj1a/foxj1b* double knockdown impaired pronephros MCC motility. Furthermore, *foxj1a* knockdown impaired ciliary motility in the ependymal cilia. In obstructed kidney tubules, expression of *foxj1a* is necessary to maintain the beating frequency of MCCs over the long term. These findings suggest a crucial role of *foxj1a* and *foxj1b* in MCC motility, with *foxj1a* playing a prominent role [224]. *gmnc* mutant displayed a reduction in the expression of *foxj1a* especially in the anterior ChP and a more homogenous reduction of *foxj1b* in the zebrafish brain. It was also found that *foxj1b* is more essential for monociliated cells in both the ChP and TC but dispensable for MCCs in these regions. In addition, double *foxj1a*^+/−^; *foxj1b*^+/−^ mutants experienced a moderate level of reduction in MCCs, and interestingly, doubly deficient *gmnc^-/-^*; *foxj1b^-/-^* exhibited a drastic loss of cilia in the TC and ChP, suggesting an important role of *foxj1a* in MCC in the ependymal cells. Therefore, it is possible that zebrafish ependymal MCCs could require both *foxj1a/b,* with *foxj1a* playing a more vital role [140].

### 7.2. Prostaglandin Signaling

Prostaglandins are a group of lipids that coordinate cell signaling via G protein-coupled receptors. They are synthesized from arachidonic acid into intermediates by cyclooxygenases (COX1, COX2), then metabolized into bioactive prostanoids such as prostaglandin E_2_ (PGE_2_) in different tissues. Prostaglandin signaling is known to mediate a wide variety of cell processes [193,225]. Two of the G protein-coupled receptors, EP2 and EP4, bind PGE_2_ and initiate a signaling cascade through cyclic adenosine monophosphate (cAMP), which activates protein kinase A (PKA). This cascade influences ciliogenesis through the intraflagellar transport of cargo proteins necessary for forming and extending cilia [194,225]. Several studies have elucidated genes that play a role in connecting prostaglandin signaling to cilia and MCC development. In zebrafish cilia development, a study showed that *lkt* encodes the ABCC4 transporter, and a mutation in this gene caused defects in ciliogenesis. The researchers also found that *lkt*/ABCC4 exports PGE_2_ and that PGE_2_ is required for proper ciliogenesis. Finally, they determined that PGE_2_ is necessary for anterograde IFT, indicating that normal cilia formation and elongation requires *lkt*/ABCC4-mediated PGE_2_ signaling [225]. Subsequently, Garcia et al. found that several PGE_2_ agonists show an ability to prevent different cilia-related renal disorders in mouse models and human urine-derived renal epithelial cells from patients with nephronophthisis, emphasizing the importance of prostaglandin signaling in cilia [226].

Recent studies further demonstrated the role of prostaglandin signaling in MCC development, where a loss of prostaglandin signaling caused a reduction in the number of renal MCC precursor cells as well as less cilia growth and basal body docking [193]. Prostaglandins were also revealed to influence the fate of zebrafish pronephros, as a lack of prostaglandin signaling caused more cells to become transporter cells instead of MCCs. Interestingly, treatment of *ets variant 5a* (*etv5a*) morphants with the prostaglandin molecule dmPGE_2_ partially rescued the MCC number. This revealed that prostaglandins function downstream of this core renal transcription factor during MCC genesis [193,227]. *ppargc1a* was found to be a key regulator of prostaglandin biosynthesis when *ppargc1a* deficient zebrafish were found to display phenotypic cilia defects and depleted MCC numbers. *ppargc1a* deficiency diminished expression of *cox1* (also known as *ptgs1*) and PGE_2_ production, where treatment with exogenous dmPGE_2_ rescued MCC number and cilia formation [194]. More recently, the transcription factor Esrrγa was discovered to interact with Ppargc1a to regulate prostaglandin signaling and thereby control MCC development in the zebrafish pronephros. The study revealed fewer MCCs and disrupted ciliogenesis in multiple zebrafish tissues, including the kidney, otic vesicle and KV. These effects were linked to disruptions in prostaglandin signaling, and the researchers found that ciliogenesis could be restored by PGE_2_ or the enzyme Ptgs1. Furthermore, the study revealed a cooperative relationship between *esrrγa* and *ppargc1a* in regulating zebrafish renal MCCs. Finally, an analysis of kidney tissue sections from mice with genetically modified *ERRγ* revealed that ERRγ is also involved in ciliogenesis in the mouse kidney, as mice lacking renal epithelial cell ERRγ exhibited shortened cilia [195,228].

### 7.3. Retinoic Acid (RA) Signaling

RA has been well studied in many developmental processes and is a major player in MCC development in zebrafish [229]. Previous work discovered that RA regulates gene expression in renal progenitors, which is essential for proximal nephron segmentation and to repress distal segment fates [156,157]. Notch signaling had also been found to be crucial in MCC cell fate choice, but its relationship to RA had not been explored [162,163]. Li *et al.* discovered that RA promotes MCC development by inhibition of the transcription factor Mecom, where Mecom promotes Notch signaling [230]. RA signaling was also found to mediate the ETS transcription factor Etv5a in regulating MCC development in zebrafish pronephros. Deficiencies of both *etv4* and *etv5a* resulted in a further decrease of MCCs, potentially suggesting that both are essential and functionally redundant. Additionally, *etv5a* acts downstream of RA signaling. Interestingly, Notch signaling was found to act upstream to inhibit *etv5a*. On the other hand, *etv4* levels in renal progenitors were not affected by changes in RA or Notch signaling [227].

In addition to Etv5a and Etv4, the Iroquois transcription factor Irx2a was discovered to be important for zebrafish pronephric MCC development downstream of RA signaling. The *Iroquois* (*iro/Irx*) genes encode a family of homeoproteins with a conserved three amino acid loop extension (TALE) domain, and they are important for patterning and specification in many tissues [231]. Interestingly, *irx2a* deficient embryos exhibited a reduction of MCCs in the pronephros. *irx2a* deficient embryos had a reduced *etv5a* expression domain, and MCC loss due to *irx2a* deficiency was rescued by provision of transcripts encoding *etv5a*. Furthermore, treatment of exogenous RA led to an expansion and a distal shift of *irx2a* expression, while blocking RA biosynthesis led to a reduction and a proximal shift of *irx2a* expression. These findings demonstrated that *irx2a* is upstream of *etv5a* and downstream of RA in promoting MCC development in the zebrafish pronephros [232].

### 7.4. Other Genes and Genetic Pathways

#### 7.4.1. miR-34/449

MicroRNAs (miRNAs) are small, non-coding RNAs that play an essential role in gene expression regulation due to post-transcriptional repression [89,233,234,235]. The miR-34/449 includes the three genomic loci, the miR-34a, miR-34b/34c and miR-449a/b/c, composed of six homologous miRNAs, namely miR-34a/b/c and miR-449a/b/c [89,236]. The microRNA-34/449 family has been recently indicated as playing important roles in MCC differentiation. Studies in other animal models found that miR-449 are strongly enriched in MCCs in human airway epithelium and *Xenopus* embryonic epidermis [88], and miR-34/449 deficient models exhibited significant decreased cilia length and number in MCC development [89]. Murine models deficient in miR-34/449 displayed dysfunctional multiciliogenesis in several tissues, such as the respiratory epithelium, female fallopian tube epithelium and male EDs, causing male infertility [121,237,238]. In the zebrafish, miR-34 regulates multiciliogenesis during organogenesis. In particular, miR-34b was found to be enriched in MCC-rich tissues such as the kidney and the nasal placode. In addition, the migration of MCCs during 51–72 hpf was delayed in embryos deficient in miR-34b compared to WTs. The study also found that cilia bundles did not form in the nasal placodes of miR-34b morphants, and cilia length was shorter compared to WTs. Additionally, miR-34b regulates multiciliogenesis through Myb. qRT-PCR found an upregulation of *myb* in *miR-34b* morphants. Meanwhile, overexpression of *myb* led to a block of cilia bundles and increased accumulation of MCCs just like *miR-34* morphants. Lastly, co-injection of *miR-34b* and *myb* morpholino rescued multiciliogenesis. These results suggest that miR-34b regulates MCC development via Myb [164].

#### 7.4.2. IFT Gene Family

IFT is an essential process for the formation and maintenance of the cilium, and many studies have demonstrated that *ift* genes play a significant role in MCC development and motility. For example, *ift70/fleer* encodes for a tetratricopeptide repeat protein in zebrafish, which is related to the *C. elegans dyf1*. *ift70* knockdown led to body curvature and pronephric cysts, indicating ciliary dysfunction. *ift70* was found to be expressed in multiple MCC tissues in zebrafish, such as in the MCCs of pronephros and the nasal placode. *ift70* mutant led to shorter cilia and disappearance of MCCs until 88 hpf, at which time the MCCs were shortened and disorganized compared to WTs. Furthermore, MCC beating amplitudes in the pronephros of *ift70* mutant were severely reduced. Additionally, nasal placode MCCs were almost absent compared to WTs [166].

In addition to *ift70*, there are other *ift* genes that have been linked to MCC development in zebrafish. For example, Ift56, also known as Ttc26, identified as an IFT complex B protein, is important for cilia development. Morpholino knockdown of *ift56* led to disruptive pronephric cilia development, and MCC beating coordination was significantly higher compared to WTs [239]. Next, *ift88* is essential for MCC development, as loss of *ift88* led to a significant loss of MCC of the nasal placode, as well as reduction of cilia in other organs such as retina and otic vesicles. Furthermore, loss of *ift57* and *ift52* leads to a significant loss of MCCs in the nasal placode. Interestingly, not all *ift* genes are essential for MCC development, as knockdown of *ift140* did not produce as significant of a loss of MCCs in the nasal placode compared to *ift88*, *ift57* and *ift52* [240]. *ift88* and *ift57* could also potentially help with pronephric MCC formation, as knockdown of both genes leads to a reduction of cilia in the pronephros [241].

#### 7.4.3. Tubulin Tyrosine Ligase-Like (TTLL) Gene Family

TTLL is a family of enzymes that plays an important role in post-translational modification of tubulin, which helps generate a variety of microtubule structures [242]. Interestingly, there is a relationship between TTLL family members and MCC development in zebrafish. *ttll* genes are expressed in several MCC tissues in zebrafish: both *ttll3* and *ttll6* are expressed in the MCCs of the pronephros, and *ttll3*, *ttll6* and *ttll7* are expressed in the nasal placode. A previous study found that *ttll6* morphants exhibit a severe reduction in MCC formation of the nasal placode compared to WTs [166]. Interestingly, *ttll3* and *ttll6* knockdown did not affect cilia length in the pronephros, but they both led to mildly dysfunctional MCC beating. Double knockdown of *ttll3/ttll6* synergistically further impaired MCC beating. Furthermore, *ttll3* morphants displayed misorientation of MCC bundles in the pronephros, suggesting a role of *ttll3* in MCC organization [243]. These studies suggest that *ttll* genes play a significant role in MCC motility in zebrafish.

#### 7.4.4. Cytoplasmic Carboxy Peptidase (CCP) Gene Family

The CCP protein family has also been investigated in its relationship to zebrafish MCC ontogeny. Previously, in situ hybridization studies found *ccp1*, *ccp2* and *ccp5* to be expressed in MCC-rich tissues such as the pronephros and the nasal placode, and *ccp1* and *ccp5* knockdown by morpholino led to disorganized and reduced size of the nasal placode [244]. A subsequent study revealed that *ccp2*, *ccp5* and *ccp6* were expressed in the nasal placode. Interestingly, *ccp5* and *ccp6* knockdown embryos had similar ciliary lengths measured by acetylated tubulin compared to WTs. However, *ccp5* knockdown led to reduced pronephric MCC beating amplitude. Interestingly, *ccp5* knockdown rescued tubulin glutamylation and promoted MCCs in *ift70/ift88* knockdown embryos [245].

#### 7.4.5. Cetn2

Centrin is a core component of the centrosome, which is required for the formation of primary and motile cilia. Cetn2, one of four centrin isoforms, was found to be essential for MCC in zebrafish [246]. MCCs of the nasal placode exhibited defects with *cetn2*-deficient embryos compared to wild-type controls. Additionally, *cetn2*-deficient embryos exhibited undetectable ciliary beating by the MCCs of the nasal placode [167].

#### 7.4.6. Ccdc57

Ccdc57 was shown to affect MCC polarity and beating in zebrafish ependymal cells. Compared to a synchronous direction of the MCCs of WT, *ccdc57* mutants exhibited MCC bending in a disorganized manner. Basal body planar polarity was absent in the *ccdc57* mutants. Additionally, cilia beating was disorganized in *ccdc57* mutants. This suggests that *ccdc57* plays an important role in MCC organization and motility [247].

#### 7.4.7. Kif6

*KIF6* is a protein of the kinesin family of motor proteins that help direct molecules to the plus-end of the microtubules [248]. *kif6*-deficient embryos developed MCC defects in the ependymal cells. Analysis of the ependymal MCCs demonstrated that *kif6* mutant zebrafish demonstrated a reduction in ependymal cilia compared to heterozygous *kif6* embryos [175].

#### 7.4.8. Ptk7

Ptk7 has been known to be an essential regulator for the Wnt-β-catenin and the non-canonical Wnt-PCP signaling pathways, and the *ptk7* zebrafish mutant has been used as a model for idiopathic scoliosis [176,249]. Interestingly, research using SEM found a significant reduction of ependymal cilia in the *ptk7* mutant compared to WTs. This suggests that *ptk7* may play an essential role in MCC development in zebrafish ependymal populations [176].

#### 7.4.9. Dyx1c1

*DYX1C1* is a candidate gene whose mutation is associated with dyslexia, one of the most common learning disabilities worldwide [250]. Using the zebrafish embryos, researchers found the *DYX1C1* ortholog gene *dyx1c1* is expressed in zebrafish MCC tissues, such as the nasal placode and the pronephros. Interestingly, *dyx1c1* morphants exhibited loss of MCCs in the nasal placode and an overall loss of pronephric cilia compared to WTs. Additionally, loss of *dyx1c1* led to loss of dynein arms in both pronephric and nasal placode cilia, suggesting an important role of *dyx1c1* in cilia motility [251].

#### 7.4.10. Yap/Taz—Co-Activators of the Hippo Pathway

Yap, an important co-activator of the Hippo pathway, was shown to affect MCC development of the zebrafish pronephros. *yap* morphants exhibited pericardial edema and cloacal cysts. At 3 dpf, pronephros MCCs did not form properly, and cilia number and length decreased significantly compared to WTs. MCCs of *yap* morphants also exhibited mislocalized basal bodies [252]. Interestingly, Taz, a paralog of Yap, did not show the same constellation of defects: *taz* morphants had fewer principal cells in the pronephric tubule, but there was no change to MCC number or ciliary beating [253]. These studies suggest that there might be a difference in the role of Yap and Taz in MCC development in zebrafish. 

#### 7.4.11. Additional Factors Potentially Regulating MCC Development

Several other genetic factors could potentially play a role in regulating zebrafish MCC formation. For example, Ccdc11, a component of the centriolar satellites, was found to be essential for MCC development in human tracheobronchial epithelial cells (hTECs), as loss of Ccdc11 led to reduced MCC numbers, and *ccdc11 Xenopus* morphants experienced shorter MCCs in their epidermis. *ccdc11* zebrafish mutant also displayed cilia defects, such as pericardial edema and dysregulated heart looping, as well as reduction of cilia length in the KV and the pronephros—data that suggest a potential role of Ccdc11 in regulating MCC development in zebrafish [254]. Another possible candidate is For20, a zebrafish homolog of the human FOR20, which acts as a pericentriolar satellite and cilium formation regulator [255]. In the zebrafish, For20 was found to be upregulated in several ciliated tissues, many of which are MCC-rich tissues such as the pronephric duct and the nasal placode. Furthermore, knockdown of *for20* led to shorter cilia in the pronephric duct, as well as disruptive cilia beating. These pieces of evidence suggest a potential role of *for20* in regulating zebrafish MCCs [256]. Next, Appa and Appb, belonging to the amyloid precursor protein (APP) family, were shown to potentially be important for MCCs of the zebrafish brain. For example, Appa and Appb are expressed in ependymal cilia of the zebrafish brain, a tissue rich in MCCs, as well as olfactory sensory neuron and otic vesicle cilia. Interestingly, cilia length in brain ventricles were longer in *appa*; *appb* double mutants compared to WTs. These findings open a promising direction in which *appa/b* are potentially essential for zebrafish MCC development [257].

## 8. Conclusions and Future Directions

In this review, we have discussed the current understanding of the genetic mechanisms that direct MCC development, focusing on recent work using the zebrafish to study renal and sensory system MCCs. We explored how multiple diseases are linked to MCC defects across organs such as the central nervous, respiratory and reproductive systems. Some conditions, such as RGMC and the global pandemic COVID-19, specifically target MCCs, thus further raising the importance of continuing to study MCC genesis. Zebrafish provide an ongoing valuable opportunity to study MCC biology due to their advantageous features for genetic studies, coupled with the abundance of MCC across accessible organs such as the pronephros. Several knowledge gaps still persist in modeling MCC-related diseases, and the zebrafish can be leveraged to address these outstanding topics. Lastly, here we provided a detailed review of the previous studies of the genetic framework that governs MCC development and motility in the zebrafish. However, there is still much potential in uncovering the link between MCCs and other signaling pathways that have been studied extensively in disease contexts [258,259]. Additionally, future studies can investigate if one gene can affect multiple MCC lineages in zebrafish, as most of the investigations reviewed here focused on one or two MCC-containing tissues. There is at present a limited understanding of how MCCs may differ structurally across species, and this will be important to address with future studies as well. With the continued emergence of new cutting-edge technologies, continuing to harness the power of zebrafish in modelling MCC diseases is likely to herald a new era of breakthrough discoveries.
